# KDM6B-mediated histone demethylation of LDHA promotes lung metastasis of osteosarcoma

**DOI:** 10.7150/thno.53347

**Published:** 2021-02-06

**Authors:** Yuhang Jiang, Fengfeng Li, Bowen Gao, Mengjun Ma, Meng Chen, Yanfeng Wu, Weidong Zhang, Yangbai Sun, Sanhong Liu, Huiyong Shen

**Affiliations:** 1 Department of Orthopedics, The Eighth Affiliated Hospital of Sun Yat-sen University, Shenzhen 518003, China.; 2 Institute of Interdisciplinary Integrative Medicine Research, Shanghai University of Traditional Chinese Medicine, Shanghai 201203, China.; 3 Department of Orthopaedics, The Second Affiliated Hospital of Guangzhou Medical University, 250 Changgang Eastern Road, Guangzhou 510260, China.; 4 Department of Musculoskeletal Surgery, Fudan University Shanghai Cancer center, Shanghai Medical College, Fudan University, Shanghai 200032, China.; 5 Department of Plastic and Reconstructive Surgery, Shanghai Ninth People's Hospital, Shanghai Jiao Tong University School of Medicine, Shanghai 200011, China.

**Keywords:** Osteosarcoma, Lung metastasis, H3K27me3 demethylation, KDM6B, LDHA.

## Abstract

**Rationale:** Osteosarcoma (OS), the most common type of bone tumor, which seriously affects the patients' limb function and life quality. OS has a strong tendency of lung metastasis, and the five-year survival rate of patients with metastatic osteosarcoma is less than 20%. Thus, new treatment targets and strategies are urgently needed.

**Methods:** The expression of the histone demethylase KDM6B and H3K27me3 levels in OS specimens were analyzed using quantitative PCR and immunohistochemical assays. The biological functions of KDM6B were determined using *in vitro* transwell, wound healing assays, and an *in vivo* orthotopic injection-induced lung metastasis model. Subsequently, chromatin immunoprecipitation sequencing (ChIP-seq) combined with transcriptomic RNA sequencing (RNA-seq), and subsequent ChIP-qPCR, western blot, and aerobic glycolysis assays were used to explore the mechanism of KDM6B function and validate the candidate target gene of KDM6B.

**Results:** KDM6B expression was significantly upregulated in OS patients, and high KDM6B expression was associated with poorer prognosis in OS patients. Targeting KDM6B significantly inhibited OS cell migration *in vitro* and lung metastasis *in vivo*. RNA-seq and ChIP-seq analysis revealed that KDM6B increases lactate dehydrogenase LDHA expression in OS cells by directly mediating H3K27me3 demethylation. The phenotypes of inhibited cell metastasis in KDM6B-knockdown OS cells was reversed upon overexpression of LDHA. Finally, a small molecule inhibitor targeting KDM6B significantly inhibited OS cell migration *in vitro* and lung metastasis *in vivo*.

**Conclusions:** Collectively, we elucidated that upregulated KDM6B facilitates tumor metastasis in OS via modulating LDHA expression. Our findings deepen the recognition of OS metastasis mechanism and suggest that KDM6B might be a new potential therapeutic target for the treatment of OS (especially highly metastatic OS).

## Introduction

Osteosarcoma (OS) is the third most common cancer in children and adolescents, and the most common type of bone tumor 1. OS is extremely harmful and seriously affects patient's limb function and quality of life 2. Thus far, the application of surgery, adjuvant chemotherapy, and neoadjuvant chemotherapy in clinical treatment has enabled the 5-year survival rate of OS patients without metastasis reached 60% - 70% 3. However, for patients with metastatic OS, the prognosis is very worrying, and the five-year survival rate is only approximately 20% or even worse 3, 4. A strong tendency for metastasis (especially lung metastasis) is an important feature of OS and metastasis is the most common cause of death. However, the molecular mechanism of OS metastasis is still unclear 5. Unfortunately, despite the increasing frequency of clinical trials with OS as an indication, the survival rate of osteosarcoma has not been significantly improved in the past 20 years, and improvements in cytotoxic chemotherapy have had very limited effect on the survival of patients with OS 6, 7. Therefore, there is an urgent need to seek new therapeutic targets and strategies for the clinical treatment of OS.

Locus-specific modifications of chromatin DNA or histones plays a very important role in the regulation of gene expression, among this histone post-translational modifications includes methylation, acetylation and ubiquitination, which affect DNA accessibility and drive differential gene expression, and then participates in the epigenetic regulation of various diseases 8. Histone 3 lysine 27 (H3K27) methylation (methylation on histone 3 lysine 27) is an important epigenetic event, methylation and demethylation of H3K27 play essential roles in many biological processes, including X-chromosome inactivation, genomic imprinting, stem cell maintenance, inflammatory response, and cancer reoccurrence 9, 10. Pharmacologic inhibition of histone H3K27 demethylation was reported to be potential treatment target for neuroblastoma and a variety of gliomas 11-13. KDM6B (lysine-specific demethylase 6B), also known as JMJD3, is a member of the JmjC histone demethylase family. KDM6B specifically demethylates trimethylated H3K27, affects chromatin accessibility and regulates gene expression by changing the chromatin configuration 14. KDM6B plays a key role in the occurrence and development of various human diseases such as cancer, immune diseases and developmental diseases 15-18. Targeted inhibition of KDM6 by a small-molecule inhibitor named GSK-J4 effectively eradicates tumor-initiating cells and downregulates stemness-associated gene signatures in colorectal cancer 19. In a previous study on epigenetic regulation of drug resistance of bone tumors, histone H3K27me3 modification was found to regulate the stemness maintenance of OS tumor stem cells 20. Given other published study suggesting that tumor stem cells are a subgroup of tumor cells that exhibit drug resistance and high metastatic potential, we wondered whether the key epigenetic enzyme KDM6B demethylating histone H3K27me3 is involved in the regulation of OS metastasis.

Malignant tumors are not only genetic diseases, but also energy metabolism diseases 21. Normal cells mainly rely on mitochondrial oxidative phosphorylation for energy supply, however, even under conditions of sufficient oxygen supply, tumor cells rely mainly on aerobic glycolysis to obtain energy, which is called the "Warburg effect" 22. Cells take up glucose through glucose transporters and use it as a substrate for glycolysis to produce pyruvate and ATP, then in cancer cells, the pyruvate is rapidly converted to lactate instead of entering mitochondria to participate in the tricarboxylic acid (TCA) cycle, as occurs in normal cells 23, 24. Therefore, cancer cells are "addicted" to aerobic glycolysis. The lactate dehydrogenase A (LDHA) protein is the enzyme responsible for catalyzing the conversion of pyruvate to lactate, and upregulation of LDHA expression has been reported to play key roles in cell growth , cell migration, cancer stem-like phenotype and chemoresistance in a variety of tumors, including lung cancer, breast cancer, pancreatic cancer, neuroblastoma and hepatocellular carcinoma 25, 26.

In our study, we found that high KDM6B expression is associated with poorer prognosis in OS patients. Depletion of KDM6B or inhibition of KDM6B enzyme activity resulted in significantly inhibited migration ability of OS cells *in vitro*, and this effect was further confirmed with an orthotopic injection model: targeting KDM6B significantly inhibited the lung metastasis of OS cells *in vivo*. We also found that KDM6B regulated LDHA transcription and OS cell aerobic glycolysis by controlling H3K27me3 demethylation in the promoter region of LDHA. Our findings revealed a new epigenetic regulatory mechanism in OS metastasis and suggested that KDM6B activity may be a potential therapeutic target for OS treatment.

## Materials and Methods

### Human tissue specimens

Human osteosarcoma tissues were collected from 63 OS patients admitted to Fudan University Shanghai Cancer Center between 2010 and 2014. Of the 63 OS patients, 12 had paracancerous tissue controls. The cancerous and paracancerous specimens were divided into two parts, one part was frozen immediately in a -80 ℃ refrigerator, the other part was fixed in 4% paraformaldehyde and subsequently embedded in paraffin for immunohistochemistry experiments. Written informed consent was obtained from all participants or their guardians (for children younger than 18 years old) before enrollment in the study. The study was approved by the Ethics Committee of Fudan University Shanghai Cancer Center, and complied with the principles expressed in the Declaration at Helsinki.

### Cell culture

The OS cell lines 143B and HOS were purchased from the American Type Culture Collection. 143B and HOS cells were cultured in DMEM supplemented with 10% fetal bovine serum (Gibco) and maintained at 37 °C in a 5% CO_2_ atmosphere.

### Transfection and lentivirus-based gene knockdown (KD) with shRNA

The FuGENE Transfection Reagent (Promega) was used for all transfections in accordance with manufacturer's instructions. For KDM6B knockdown, a KDM6B-specific shRNA was cloned into the pLVX-shRNA1 plasmid (Clontech). KDM6B-KD1: GTGGGAACTGAAATGGTATTT, KDM6B-KD2: GATGATCTCTATGCATCCA. For LDHA knockdown, LDHA-KD1: CAATCTGGATTCAGCCCG, LDHA-KD2: GCAAACTCCAAGCTGGTC. For a negative control, a scrambled sequence was used. Packaging was conducted with a three plasmid-system with psPAX2 and pMD2G (Clontech). The lentiviral supernatant was used to infect OS cell lines, stable cell lines were obtained after two weeks of screening with 2 μg/mL puromycin.

### RNA extraction, RNA-seq and RT-quantitative PCR (qPCR)

Total RNA was extracted from OS tissues or cells using TRIzol reagent (Invitrogen) according to the manufacturer's instructions and then treated with DNase I (Promega). For the RNA-seq, total RNA from control and KDM6B-KD 143B cells was subjected to HiSeq RNA-Seq performed by BGI Tech Solutions Co. The cDNA libraries were sequenced using an Illumina HiSeq2000. Transcriptome reads from RNA-Seq experiments were mapped to the reference genome (hg19) using the Bowtie tool. Gene expression levels were quantified by the software package RSEM (https://deweylab.github.io/RSEM/). The list of significance was operated by setting of a P value threshold at 0.05. The differentially expressed genes were subsequently analysed for enrichment of biological terms with the Database for Annotation, Visualization and Integrated Discovery (DAVID, david.abcc.ncifcrf.gov) bioinformatics platform. The RNA-seq data that support the findings of this study have been deposited in SRA database (NCBI) and are accessible through accession number PRJNA668543. For the RT-qPCR assay, reverse transcription of 1 μg of total RNA was performed to obtain complementary DNA (cDNA) using Transcript First Strand Synthesis Supermix (TransGen). qPCR was performed using a 7500 Fast Real-Time PCR System (Applied Biosystems) and SYBR Green Supermix (TaKaRa) following the manufacturer's protocol. Raw data were normalized to GAPDH as the internal control and are presented as the relative expression levels, which were calculated as relative quantification (RQ) = 2^-ΔΔCt^. All primers for RT-qPCR are listed in Table S1.

### Western Blot

Loading lysis buffer (Beyotime) was used to extract total protein from cells. The Lowry protein assay was then used for protein level quantification. 30 mg of protein lysate were then run on an SDS/PAGE gel and transferred to a PVDF membrane (Millipore), blocked with 5% nonfat milk, incubated with appropriate primary and HRP-conjugated secondary antibodies (Cell Signaling Technology, 7074). Antibodies used in this study were: H3K27me3 (Active Motif, 39155), KDM6B (Cell Signaling Technology, 3457), LDHA (Cell Signaling Technology, 3582), ACTIN (Sigma-Aldrich, A5441). Antibody binding was detected using SuperSignal West Pico Chemiluminescent Substrate (Pierce) and photographed by ImageQuant™ LAS 4000.

### Chromatin immunoprecipitation (ChIP)-seq and ChIP-qPCR

OS cells were cross-linked with 1% fresh formaldehyde for 10 min at room temperature, neutralized with glycine for 5 min and lysed in SDS lysis buffer. The cross-linked DNA was then sheared into fragments ~200-1000 bp in length with UCD-300 (Bioruptor). ChIP was performed with a Chromatin Immunoprecipitation Kit according to manufacturer's instructions (Millipore, 17-371) to obtain ChIP-enriched DNA. ChIP-Seq assay was performed as we had previously described 27, the 75-nt sequence reads generated by Illumina sequencing were mapped to the genome using the Burrows-Wheeler Aligner algorithm with default settings. Only reads that passed Illumina purity filter, aligned with no more than 2 mismatches, and mapped uniquely to the genome were used in the subsequent analysis. Peak calling was done by MACS (version 2.1.0). The final overlapped peaks across replicates were determined as the H3K27me3 enriched regions. Average of peak values of all active regions in the gene and within the gene margin were used to calculate the differentially enriched genes. The ChIP-seq data that support the findings of this study have been deposited in SRA database (NCBI) and are accessible through accession number PRJNA668695. For the ChIP-qPCR assay, subsequent qRT-PCR was performed to quantify the ChIP-enriched DNA. The data were normalized to the input. The antibodies used for ChIP were anti-H3K27me3 (Active Motif, 39155). ChIP-qPCR primers sequence were listed in Table S2.

### CCK-8 assay

The Cell Counting Kit-8 (Beyotime) was used in accordance with provided protocols. Briefly, 2000 cells/well were added to a 96-well plate, 10 µL CCK-8 solution was added per cell, then cells were incubated for 2 h at 37 ℃, and absorbance at A450 was read at Thermo Scientific Microplate Reader.

### Flow cytometry

KDM6B-KD and control OS cells were plated in a 6 well plate, for cell apoptosis analysis, cells were collected and stained in 100 µL of staining buffer (PBS + 0.5% bovine serum albumin (BSA)) containing 7AAD (BD Biosciences) and Annexin V-APC (BD Biosciences). After incubation on ice for 30 min, the cells were washed with PBS and collected with FACS Calibur (BD Biosciences), and the data were analyzed using FlowJo software.

### Wound healing assay

Cells were plated in 6-well plates and grown until they reached confluence. Cell monolayers in 6-well plates was artificially scratched with 10 μL pipette tips. The wounded cell monolayer was washed and the wound areas were photographed under a microscope 0 and 12 h after scratching and measured using a caliper. Cell mobility was defined as the percentage of repaired area and calculated using the following formula: (1-(current wound size/initial wound size)) × 100.

### *In vitro* invasion assay

*In vitro* invasion assays were conducted using transwell inserts (Costar) containing 8-μm pore-size polycarbonate membrane filters in 24-well culture plates. 1 × 10^5^ 143B or HOS cells in serum-free DMEM were seeded in the upper chamber, coated with Matrigel (Becton Dickinson) at the bottom. The lower chamber was flooded with 10% FBS DMEM. After 12 h, cells that migrated to the lower chamber were fixed with 4% polyoxymethylene and stained with 0.05% crystal violet (Sigma-Aldrich). The migrated cells were imaged and counted in 3 random fields.

### *In vivo* metastasis experiments

For the *in vivo* osteosarcoma lung metastasis model, 6-week-old BALB/c nude mice were purchased from Guangdong Experimental Animal Center and bred in specific pathogen-free (SPF) conditions. 1× 10^6^ OS 143B cells in 10 μL PBS were injected orthotopically into medullary cavity of tibia of mice. After 3 weeks, mice were sacrificed and the lungs were collected and fixed with Bouin's solution to count the number of metastatic pulmonary nodules, then lungs were embedded in paraffin for subsequent hematoxylin and eosin (H&E) staining. For the *in vivo* imaging, we used 143B-luci cells stably expressing luciferase, and luciferase substrate D-Luciferin was retro-orbitally injected before imaging at day 7, 14, 21 and 23. The animal experiments were performed in strict accordance with the “Guide for the Care and Use of Laboratory Animals” and were approved by the Ethics Committee of The Eighth Affiliated Hospital of Sun Yat-sen University.

### Measurement of intracellular glucose uptake, LDH activity, and lactate production

Glucose uptake was measured with a Glucose Colorimetric Assay Kit II (BioVision, K686) according to the manufacturer's instructions. A standard glucose calibration curve was prepared under the same conditions to calculate the glucose uptake of cell samples. LDH activity were measured using Lactate Dehydrogenase Activity Assay Kit (Sigma-Aldrich, MAK066), 1 × 10^6^ 143B or HOS cells were homogenized on ice in 500 µL of cold LDH Assay buffer and centrifuged with 10000 × g for 15 min at 4 °C. The absorbance at 450 nm was measured by spectrophotometric multi-well plate reader. Lactate production was detected by Lactate Assay Kit (Sigma-Aldrich, MAK064), the procedure was performed as recommended by the manufacturer. Cells were homogenized in Lactate Assay Buffer and centrifuged with 13,000 × g for 15 min at 4 °C. The absorbance at 570 nm was measured.

### Statistical analyses

All experiments were performed in triplicate unless stated otherwise, and data were presented as mean ± standard deviation. Statistical analyses were performed using R (http://www.r-project.org/), and statistical significance was determined by two-tailed Student's t-test or Spearman correlation coefficients test. For all statistical tests, A P value < 0.05 was considered significant (*: *P* < 0.05).

## Results

### KDM6B is upregulated in metastatic OS and associated with prognosis

**Figure 1:**
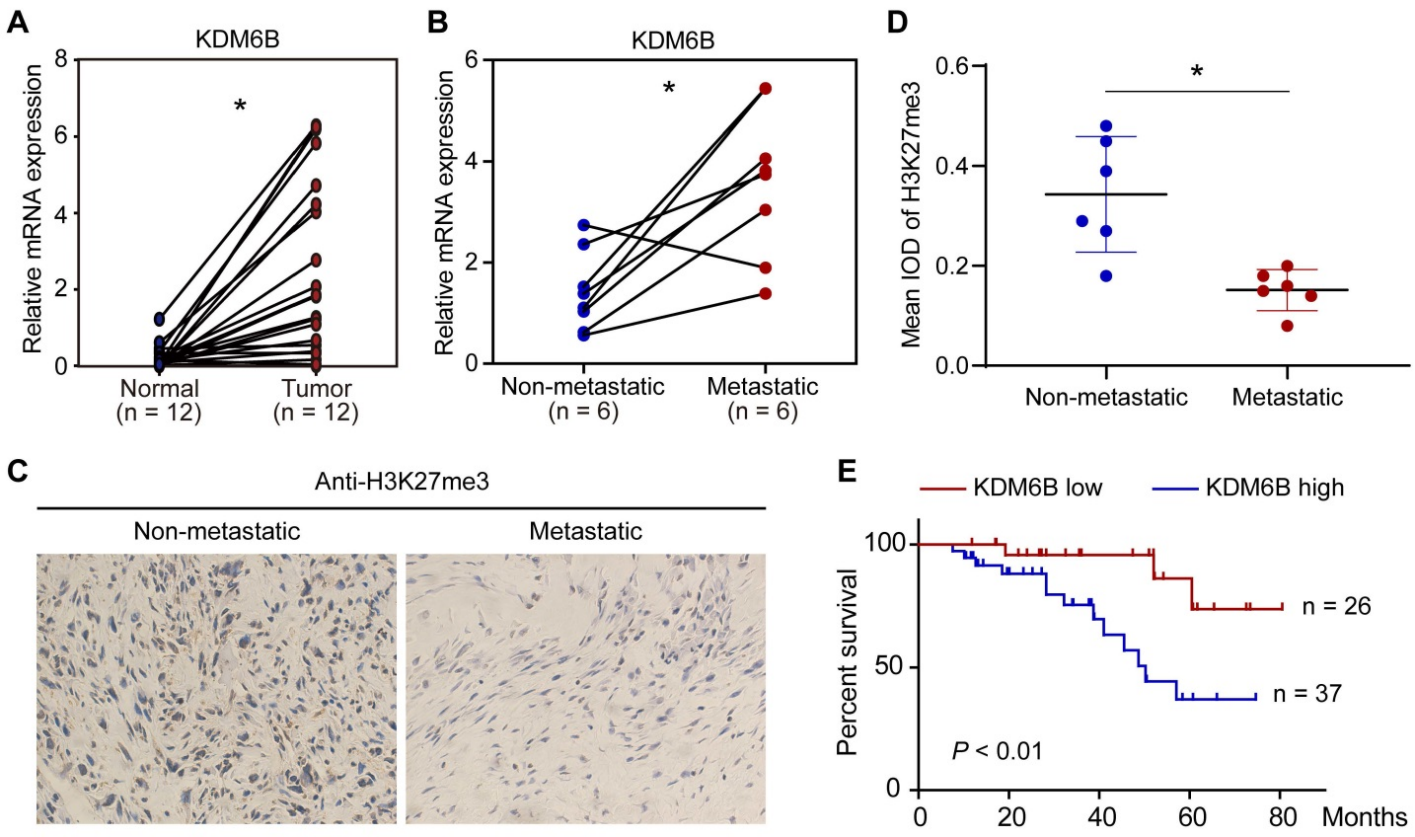
** KDM6B expression is upregulated in highly metastatic OS. (A)** Expression of KDM6B in 12 pairs of primary OS patients samples as determined by RT-qPCR. **(B)** KDM6B mRNA expression in patients with metastatic and non-metastatic OS. **(C)** Representative images of immunohistochemical staining for H3K27me3 in OS patients described in (B). **(D)** Quantitative analysis of IHC results in (C). **(E)** Kaplan-Meier plot of overall survival of 63 OS patients based on KDM6B expression levels (KDM6B high: blue line, KDM6B low: red line). A log-rank test was used for statistical analysis. *P < 0.05.

To explore a possible role of KDM6B-mediated H3K27me3 demethylation in OS, we first compared the expression of histone demethylase KDM6B in 12 pairs of tumor tissues and their corresponding nontumor counterparts. The mRNA of tissue samples was extracted for RT-qPCR analysis, results indicated that KDM6B mRNA was significantly increased in tumors compared with their paired normal biopsies (Figure 1A). The OS patients were divided into a metastatic group and a non-metastatic group. We found that the KDM6B mRNA expression was significantly upregulated in patients with metastatic OS (Figure 1B). In line with this observation, immunohistochemistry (IHC) was performed using H3K27me3 antibody, and it was found that the level of H3K27me3 modification in the tumor specimens of metastatic osteosarcoma patients was significantly reduced in patients with metastatic OS (Figure 1C-D). Further analysis of survival data revealed that patients with high KDM6B expression (> median) were significantly associated with poorer overall survival, suggesting KDM6B expression levels might serve as an independent predictor for risk stratification of overall survival in OS patients (Figure 1E). These findings highlight KDM6B as a potential biomarker for OS diagnosis and suggest a causal role of KDM6B in OS metastasis.

### Targeting KDM6B significantly attenuated OS cell migration ability *in vitro*

**Figure 2:**
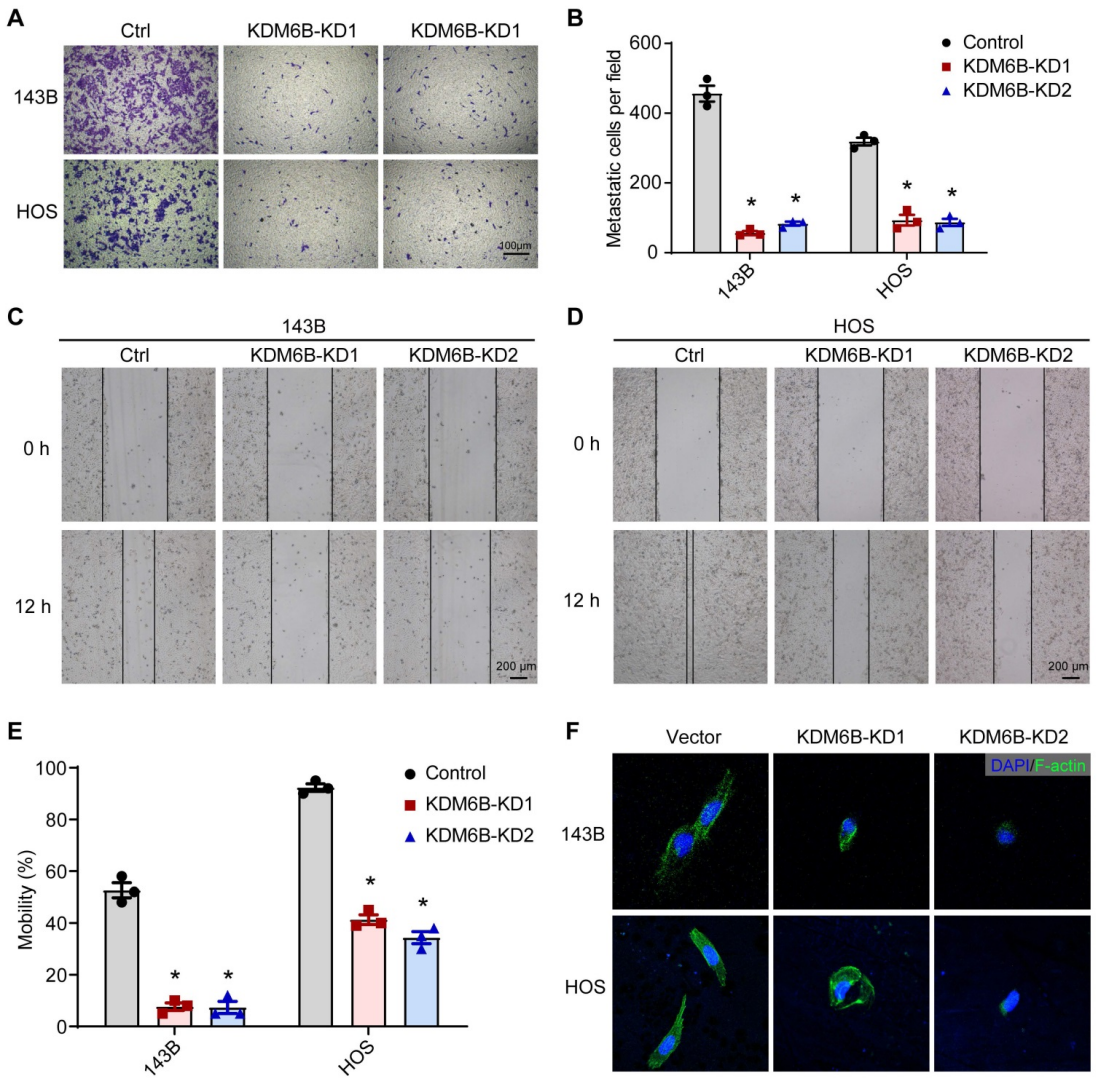
** KDM6B promotes osteosarcoma cell migration *in vitro*. (A)** *In vitro* migration of control (Ctrl) and KDM6B-knockdown (KDM6B-KD) 143B and HOS cells as assessed by transwell assay. **(B)** The quantitation results of (A). **(C**-**D)** Wound-healing assay of control and KDM6B-KD 143B and HOS cells. **(E)** Quantitative results for (C, D). **(F)** Control/KDM6B-KD 143B and HOS cells were stained with phalloidin and photographed under a confocal laser microscope. *P < 0.05.

To define the role of KDM6B in OS metastasis, we first generated KDM6B-depleted OS cells. We used the lentiviral vector-mediated shRNA knockout strategy to knock down the expression of KDM6B in OS cell lines 143B and HOS. The CCK8 assay and flow cytometry results showed that KDM6B did not significantly affect cell growth and apoptosis (Figure S1A-D). Then we examined the migration ability of OS cells *in vitro* after KDM6B knockdown by transwell experiments. KDM6B knockdown (KDM6B-KD) significantly inhibited the migration ability of two OS cells *in vitro* (Figure 2A- B). Wound-healing assay also showed that 143B and HOS cells with KDM6B depletion or inhibition significantly exhibited reduced cell migration compared with control cells (Figure 2C-E). Moreover, we observed morphological changes and altered actin cytoskeleton in KDM6B-KD OS cells, such changes might affect epithelial-mesenchymal transition, a critical event that occurs during carcinoma invasion and metastasis (Figure 2F). Together, these results suggested that KDM6B inhibition significantly inhibited migration and invasion of OS cells *in vitro*.

### KDM6B promotes lung metastasis of OS cells *in vivo*

**Figure 3:**
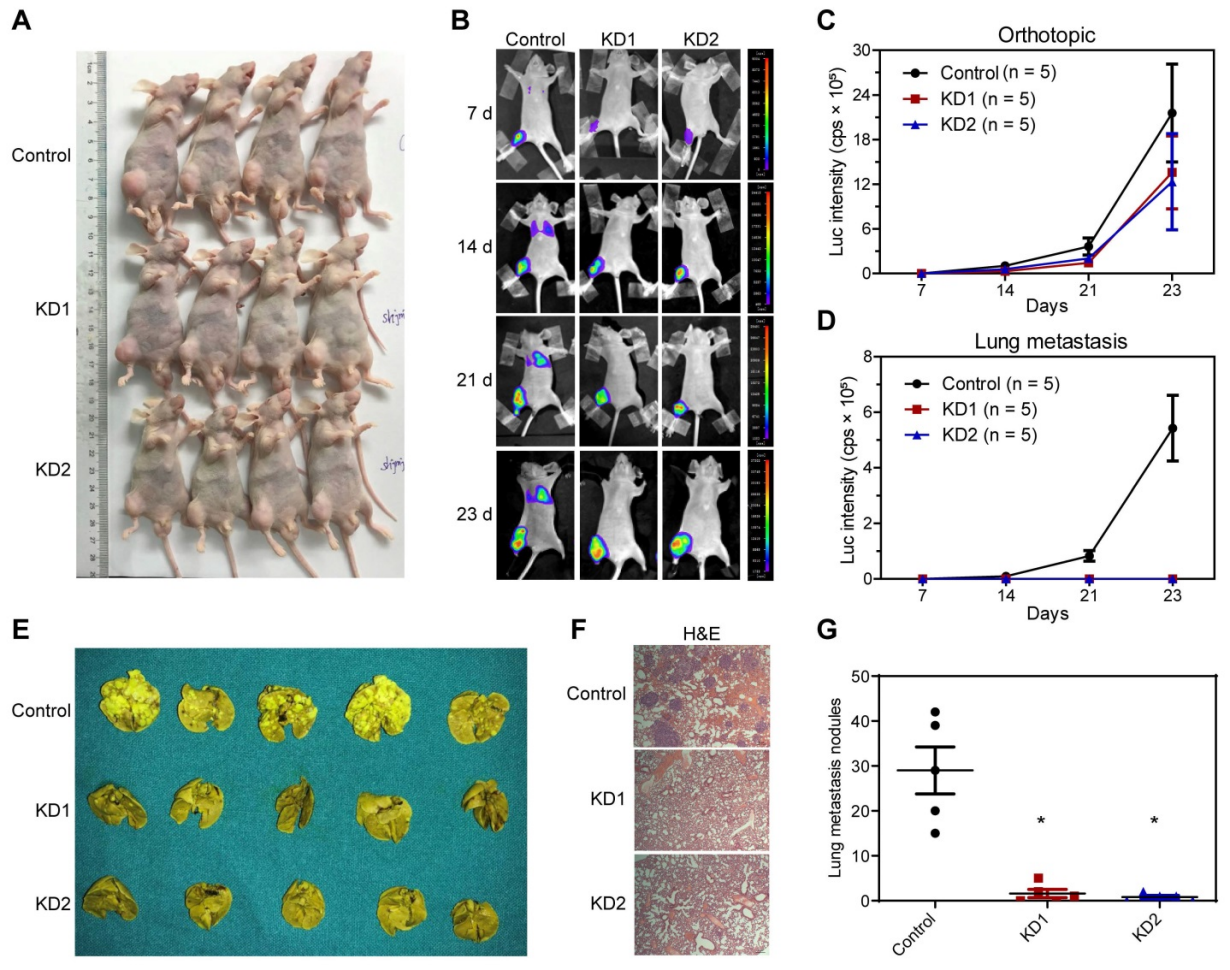
** Silencing KDM6B inhibited lung metastasis of OS in nude mice models. (A)** General view of the posterior limb with tumors. **(B)** Representative images at different timepoints showed the progression of orthotopic tumor and lung metastasis in control and KDM6B-KD groups as examined by *in vivo* bioluminescent imaging. **(C**-**D)** Bioluminescence intensity quantification of orthotopic tumors and lung metastasis (n = 5 per group). **(E)** Lung tissues in control and two KDM6B-KD groups were excised at day 23 and fixed with Bouin's solution. **(F)** Representative images of lung tissues subjected to H&E staining in control and two KDM6B-KD groups. (**G**) Lung metastasis nodules were counted manually. *P < 0.05.

Next, we constructed an *in vivo* lung metastasis model of OS by injecting 143B cells labeled with luciferase expression into the medullary cavity of tibia in nude mice. *In vivo* imaging after injection with luciferase substrate D-Luciferin was used to detect the progress of orthotopic tumor and lung metastasis. As revealed by the general view and bioluminescent imaging (BLI), there was no significant difference in orthotopic tumorigenesis (Figure 3A-C and Table S3). According to the BLI and measurement of luciferase intensity, control 143B cells profoundly metastasized to the lung after 2 and 3 weeks of inoculation, whereas KDM6B-KD1 and KDM6B-KD2 groups (injected KDM6B-KD 143B cells with different shRNA sequences) displayed significantly weaker luminescent signals, practically no obvious lung metastases were observed in two KDM6B-KD groups (Figure 3B-D and Table S3). To substantiate these observations, lung tissues were taken from the sacrificed mice and fixed with Bouin's solution, then H&E histochemistry was performed, and the results indicated that the number of metastatic pulmonary nodules was significantly reduced in KDM6B-depletion groups than in the control group (Figure 3E-G). Overall, these results indicated that KDM6B depletion significantly inhibited the lung metastasis of osteosarcoma cells *in vivo*.

### Transcriptome sequencing and ChIP sequencing identified LDHA as a KDM6B downstream target

**Figure 4:**
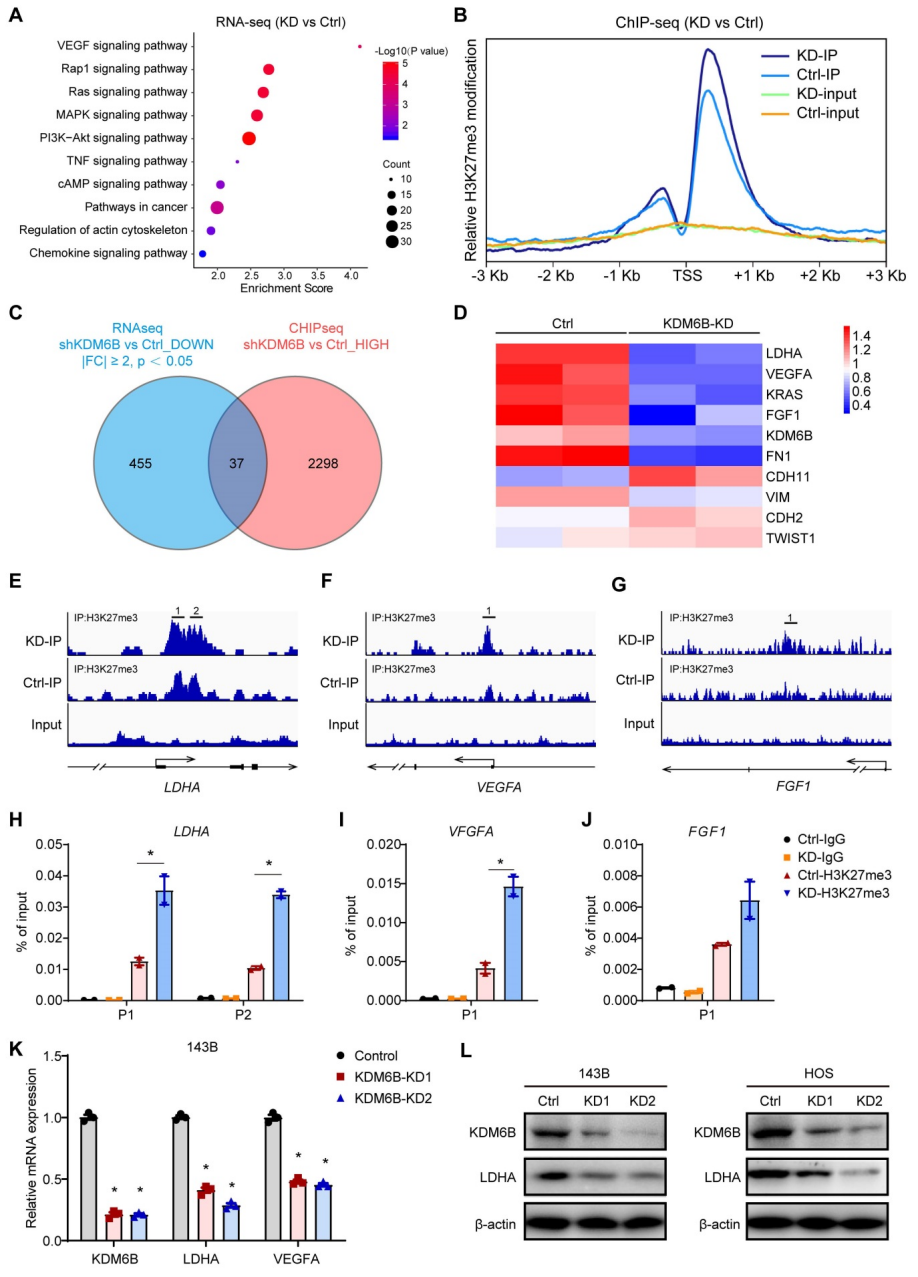
** KDM6B promotes LDHA expression through direct regulation of H3K27me3 demethylation. (A)** Gene Ontology analysis of differentially expressed genes in KDM6B-KD 143B cells compared with control 143B cells. **(B)** Tag density profile of H3K27me3 distribution of genes showing the decrease in H3K27me3 modifications in KDM6B-silenced OS cells. **(C)** Venn diagram indicating the overlap of genes with reduced expression levels (left, blue) and increased H3K27me3 modification levels (right, red) in KDM6B-knockdown cells. **(D)** Heat map summarizing the RNA expression of relevant overlapping genes in (C). **(E-G)** Snapshot of H3K27me3 ChIP-Seq signal at the represented gene locus in control and KDM6B-KD OS cells. **(H-J)** Results of ChIP-qPCR to confirm changes in H3K27me3 modification near the TSS of the indicated genes in control and KDM6B-KD OS cells. **(K)** Determination of KDM6B, LDHA, VEGFA mRNA expression levels by RT-qPCR in control/KDM6B-KD 143B and HOS cells. **(L)** Immunoblots for KDM6B and LDHA expression in control/KDM6B-KD 143B and HOS cells. *P < 0.05.

To delineate the functional implications of KDM6B and identify its downstream targets in OS, we performed transcriptome sequencing to interrogate the expression changes in KDM6B-knockdown cells. Kyoto encyclopedia of genes and genomes (KEGG) pathway analysis revealed that differentially expressed genes were significantly enriched in gene sets involved in VEGF signaling, Rap1 signaling and Ras-MAPK signaling, suggesting that KDM6B might have profound impacts on cancer biology (Figure 4A). As KDM6B was a histone demethylase that mainly mediates H3K27me3 demethylation, to determine whether KDM6B regulates the expression of key genes involved in tumor metastasis by the activity of H3K27 demethylase, we conducted ChIP-Seq (chromatin immunoprecipitation followed by high-throughput sequencing) to characterize the differences in the genomic distribution of H3K27me3 in control and KDM6B-KD OS cells. Comparison of ChIP-Seq profiles in control and KDM6B-KD OS cells revealed a total of 2298 genes displaying increased H3K27me3 levels after KDM6B silencing (Figure 4B-C). In line with the notion that histone H3K27 modification is associated with gene transcription repression, the difference in the peaks between control and KDM6B-KD cells was more obvious within the promoter areas and gene bodies in a region of approximately 2 kilobases around the transcription start site (TSS) (Figure 4B). To further correlate chromatin bindings with direct gene regulation, we integrated the transcriptome sequencing with ChIP-Seq data and noticed that a total of 37 genes might be directly regulated by KDM6B, as they showed reduced expression levels and increased H3K27me3 modifications in KDM6B-KD OS cells (Figure 4C). Among these genes, we selected lactate dehydrogenase A (LDHA) as a candidate target gene of KDM6B-mediated H3K27me3 modification for further investigation (Figure 4D). An H3K27me3 peak was detected around the TSS of LDHA mRNA in control OS cells and was significantly increased upon KDM6B knockdown (Figure 4E). Similar H3K27 modification level change was also found in the LDHA upstream gene VEGFA and FGF1 (Figure 4F-G). These histone methylation changes were further validated using ChIP-quantitative PCR (Figure 4H-J). The results of RT-qPCR and western blot in 143B cells and HOS cells also further verified the decreased LDHA expression after KDM6B knockdown at RNA and protein levels (Figure 4K-L). These data indicated that reduced LDHA expression in KDM6B-KD OS cells was associated with H3K27me3 accumulation, so LDHA was identified as a KDM6B downstream target.

### LDHA reverses the phenotype mediated by KDM6B depletion in OS

**Figure 5:**
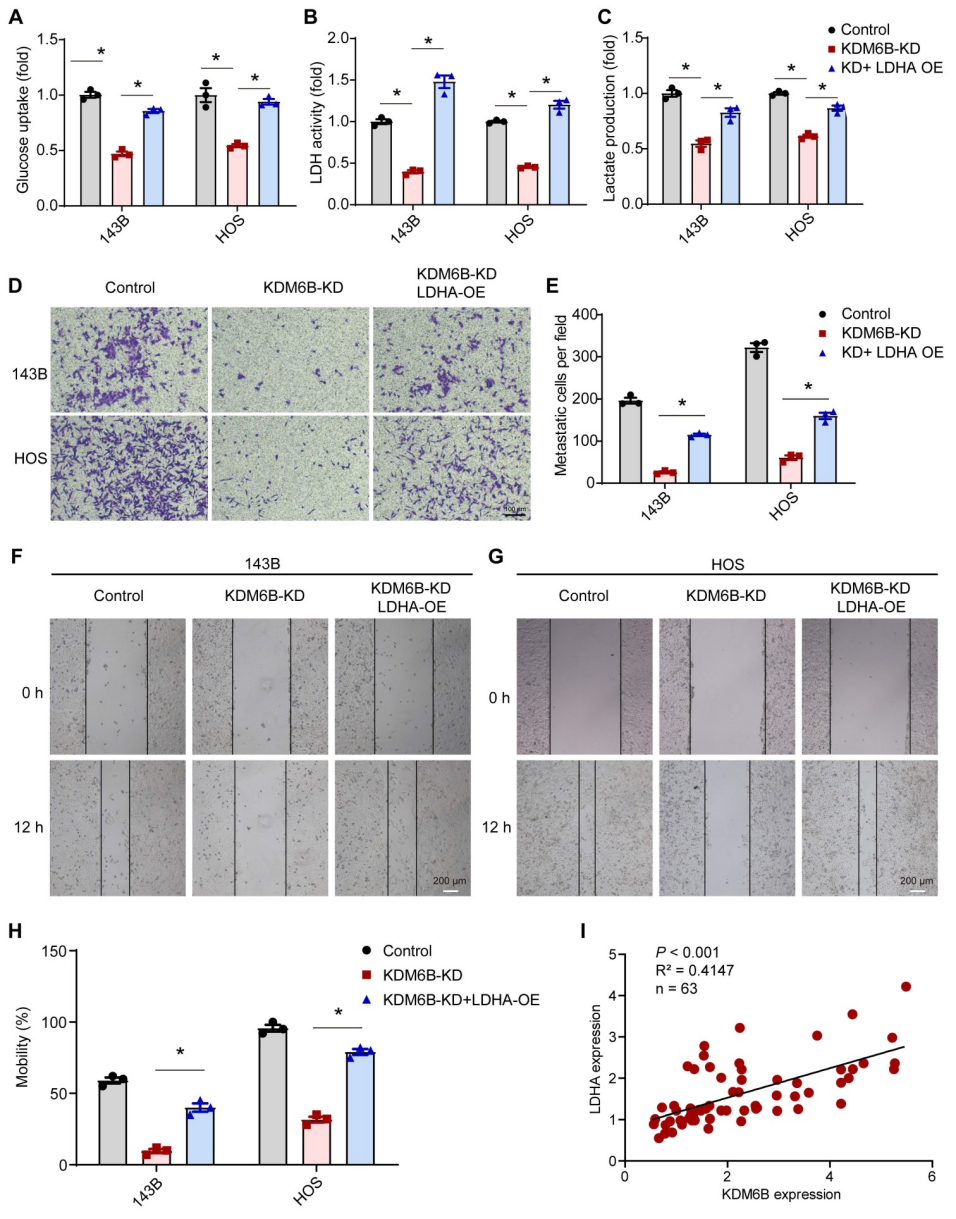
** KDM6B knockdown inhibited glycolysis and LDHA overexpression rescue the inhibitory effect of KDM6B-KD on OS cell migration. (A-C)** Glucose uptake, LDH activity, and lactate concentrations were measured in control/KDM6B-KD 143B and HOS cells as described in Materials and Methods. **(D)** *In vitro* migration of control, KDM6B-KD and KDM6B-KD with LDHA-overexpression (OE) 143B or HOS cells as determined by transwell assay. **(E)** Quantitation results for (D). **(F**-**G)** Wound-healing assay of control, KDM6B-KD and KDM6B-KD+LDHA-OE143B or HOS cells. **(H)** Quantitative results for (F, G). **(I)** The mRNA expression of KDM6B and LDHA in OS tissue specimens was analyzed by Spearman rank correlation coefficient analysis. *P < 0.05.

As LDHA executes aerobic glycolysis in tumor cells by catalyzing the conversion of pyruvate to lactate, to determine the metabolic effects of KDM6B silencing in OS cells, we calculated the aerobic glycolysis index in control and KDM6B-KD OS cells. Knockdown of KDM6B lead to significantly decreased LDH activity, glucose utilization, and lactate concentrations in 143B cells, and this inhibitory effect was attenuated upon overexpression of LDHA (Figure 5A-C). LDHA expression in OS tissues was identified to be upregulated compared with para-tumor tissues, and LDHA mRNA expression was significantly up-regulated in patients with metastatic osteosarcoma (Figure S2A-B). Then we generated LDHA-depleted OS cells to assess the role of LDHA in OS metastasis (Figure S2C), we found that LDHA knockdown (LDHA-KD) also significantly inhibited the migration ability of 143B and HOS cells, similar as the significant effect of KDM6B depletion on OS cell migration (Figure S2D-F). Further analysis revealed that lower level of LDHA expression implied better prognosis according to the overall survival analysis (Figure S2G). Moreover, we performed overexpression of LDHA in KDM6B-KD OS cells to examine whether LDHA was able to reverse the phenotypes (Figure S2H). LDHA overexpression was sufficient to partially rescue the inhibitory effect of KDM6B knockdown on cell migration as demonstrated by transwell and wound-healing assay results (Figure 5D-H). More importantly, LDHA expression was positively correlated with KDM6B expression in our OS cohort (Figure 5I). Hence KDM6B might function in OS metastasis via the KDM6B-LDHA axis.

### KDM6B inhibitor treatment suppresses OS cell migration and lung metastasis

**Figure 6:**
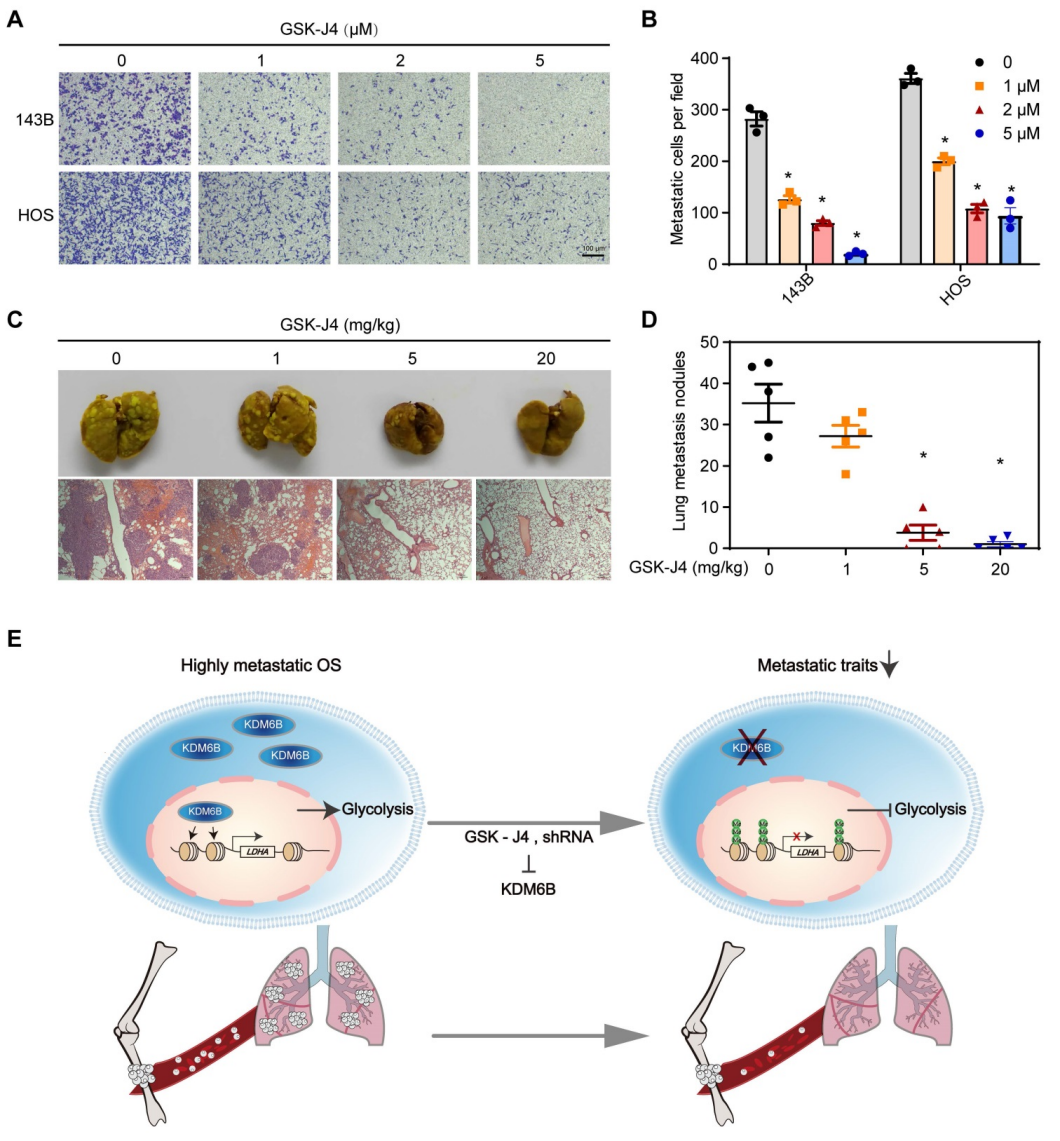
** Small molecule inhibitor of KDM6B suppresses OS cell migration *in vitro* and lung metastasis *in vivo*. (A)** *In vitro* migration of 143B and HOS cells treated with various concentrations of GSK-J4 as determined by transwell assay. **(B)** Quantitation results for (A). **(C)** Representative images of bouin's solution-fixed lung tissues in control and GSK-J4-treatment groups. 6 μm sections of lung tissues were subjected to H&E staining (lower panel). **(D)** Lung metastasis nodules of the control and GSK-J4-treatment groups were counted manually. *P < 0.05. **(E)** Working model diagram of how KDM6B regulates metastasis in OS.

GSK-J4 is a cell-permeable and selective small molecule inhibitor of KDM6B, which has approximately above 10-fold lower activity against KDM5A/B than other JmjC demethylases *in vitro* 28, 29. GSK-J4 has been used for selective pharmacological blockade of KDM6B in various mouse disease models 12, 17. We found that GSK-J4 treatment resulted in significantly inhibited OS cell migration *in vitro*, similar as the effect of KDM6B depletion (Figure 6A-B). Moreover, in the *in vivo* lung metastasis model where osteosarcoma cells were injected into the medullary cavity of nude mice, intraperitoneal administration of GSK-J4 to concentrations above 5 mg/kg was able to significantly inhibit the lung metastasis of OS cells *in vivo* (Figure 6C-D). These results strongly suggested the application prospect of KDM6B as a treatment target for highly metastatic OS.

## Discussion

As a highly metastatic malignant tumor, poor prognosis of lung-metastatic OS patients is a key issue in the treatment of OS. Our results highlighted that histone demethylase KDM6B played an oncogenic role in the progression of osteosarcoma, KDM6B-mediated H3K27me3 demethylation promotes oncogenic LDHA expression, thereby facilitating OS cell migration *in vitro* and lung metastasis *in vivo* (Figure 6E).

So far, epigenetic regulation has been found to play important roles in tumorigenesis, metastasis, cancer cell stemness, and drug resistance in various cancer types, but importance of epigenetic enzymes that controlling OS metastasis is rarely understood. LDHA uncovers a link between glycolysis and tumor maintenance, and is considered as one of the leading genes that promote tumorigenic potential of malignancies 30, 31. Inhibition of LDHA suppresses tumor growth and metastasis, reduces malignant transformation in a number of malignancies, indicating an important role for LDHA in tumor initiation and progression 26, 32, 33. We believe that the regulation of LDHA gene expression was an important but perhaps not the only target of KDM6B in the regulation of OS lung metastasis, whether KDM6B also regulates other important genes associated with OS tumorigenesis and progression still needs to be determined.

KDM6B epigenetically regulates development and differentiation, tumorigenesis, adaptive immunity, and various human diseases by demethylating chromatin marks H3K27me3, but a role for KDM6B in metabolic regulation has not been widely described. It is reported in the study of osteoarthritis (OA) that KDM6B controls the balance between anabolic and catabolic metabolism in chondrocytes, KDM6B ablation accelerates osteoarthritis development through suppression of anabolism 34. KDM6B epigenetically activated mitochondrial β-oxidation during fasting, Liver-specific KDM6B depletion lead to hepatosteatosis as well as glucose and insulin intolerance 35. Moreover, owing to the H3K27 demethylases KDM6A and KDM6B were identified as central regulators of T helper (Th) cell differentiation, studies showed that GSK-J4 increases genome-wide levels of the H3K27me3 chromatin mark and impacts on processes comprising metabolic pathways, such as ATP synthesis, the citric acid cycle, *etc*., leading to suppression of RORγt during Th17 differentiation 36. There is also a preliminary study suggesting KDM6B might mediate sodium lactate-induced glycolysis so as to enhance human MSC stemness 37. In our study, we found that KDM6B regulates glycolysis in tumor cells via modulating lactate dehydrogenase A expression, and then affect the progress of OS. Our research would further expand the understanding with regard to metabolic role of KDM6B, uncovering its special and important function in lung metastasis of OS.

Recent studies have revealed the mechanism and application value of ferroptosis in tumor treatment 38-40. Schreiber *et al.* reported that inhibiting GPX4 with small molecule compounds could induce ferroptosis in drug-resistant cancer cells in the interstitial state, thereby killing tumor cells with high EMT status and inhibiting tumor metastasis, these findings suggest that ferroptosis plays an important role in the process of tumor EMT and metastasis 41. Therefore, targeting ferroptosis may be a very promising new method for the treatment of tumor metastases derived from mesenchymal cells including OS. In our study we noticed that several ferroptosis-related genes such as *Sla7a11* and *Ptgs2* (data not shown), were among the genes that were significantly differentially expressed after KDM6B KD in OS cells, there are so we wonder whether KDM6B also regulates lipid peroxidation and ferroptosis process in highly metastatic OS cells, so as to affect the EMT process. EMT is a critical event that occurs in carcinoma invasion and metastasis 42. It has been reported that overexpression of KDM6B in epithelial ovarian cancer cells promoted EMT by inducing transforming growth factor‐β1 (TGF-β1) 43, 44. What's more, KDM6B played a permissive role in TGF-beta-induced EMT in mammary epithelial cells by stimulating SNAI1 expression 45. However, in our study no significant change in TGF-β1 or SNAIL expression was detected in KDM6B-KD OS cells. which might indicate the existence of dissimilar regulatory mechanisms in different tumors. All these related issues deserve further exploration.

Though we have demonstrated the oncogenic role of KDM6B in OS cell metastasis, we didn't determine which abnormal conditions led to elevated KDM6B expression in OS. Under normal conditions, the expression level of KDM6B in tissues is low, but the activation of many inflammatory or cellular stress signals can strongly induce the expression of KDM6B, such as MAPK signaling, NF-κB signaling, Wnt signaling and some growth factor signaling 46, 47. Therefore, on one hand, we speculate that these constitutively activated inflammatory signals might be a cause of the high KDM6B expression in OS. On the other hand, in our study we observed that the expression of upstream signaling molecules such as VEGFA and FGF1 was also regulated by KDM6B, which may form a positive feedback loop, strengthening the inhibition of tumor progression after targeted blockade of KDM6B.

Besides, histone demethylases have also been reported to be involved in the modification of non-histone protein. For instance, the H3K9me2/3 demethylase JMJD2D was able to promote liver cancer initiation and progression independent on its demethylase activity, instead, JMJD2D directly interacted with p53 and inhibited p53 recruitment to the p21 and PUMA promoters, implicating a demethylase-independent function 48. Whether KDM6B also plays other H3K27me3 demethylase-independent functions still needs to be further explored.

## Conclusions

In summary, our work demonstrates the key function of elevated KDM6B in OS metastasis. This study elucidates a new epigenetic regulation mechanism in which KDM6B regulates tumor metastasis by directly mediating H3K27me3 demethylation of the glycolysis-related gene LDHA, thereby leading to increased LDHA expression and facilitated tumor metastasis in OS. Our study suggested that targeting KDM6B might be a new approach for therapeutic OS treatment to be achieved.

## Supplementary Material

Supplementary figures and tables.

## Abbreviations

OS: Osteosarcoma
KDM6B: Lysine-specific demethylase 6B
ChIP-seq: Chromatin immunoprecipitation sequencing
RNA-seq: RNA sequencing
H3K27me3: The tri-methylation on histone 3 lysine 27
LDHA: Lactate dehydrogenase A
CCK-8: Cell Counting Kit-8
FACS: Fluorescence-activated cell sorting
H&E: hematoxylin and eosin
SPF: Specific pathogen-free
RT-qPCR: Quantitative reverse transcription polymerase chain reaction
KEGG: Kyoto encyclopedia of genes and genomes
Ctrl: control
KD: Knockdown
OE: Overexpression
TSS: transcription start site
EMT: epithelial-mesenchymal transition
HR: Hazard ratio
