# Supplementary material for: KDM6B-mediated histone demethylation of LDHA promotes lung metastasis of osteosarcoma
Source: Theranostics. 2021 Feb 6;11(8):3868–81. doi: 10.7150/thno.53347 (PMC7914357; doi:10.7150/thno.53347)
Supplement: Supplementary file 1 — Supplementary figures and tables. [file thnov11p3868s1.pdf]

Supplementary materials for

KDM6B-mediated histone demethylation of LDHA promotes lung metastasis of osteosarcoma

Yuhang Jiang[1*], Fengfeng Li[3*], Bowen Gao[5*], Mengjun Ma[1], Meng Chen[1], Yanfeng Wu[1], Weidong Zhang[2], Yangbai Sun[4#], Sanhong Liu[2#] and Huiyong Shen[1#]

Correspondence authors: Yangbai Sun, E-mail: drsunyb@fudan.edu.cn; Sanhong Liu, E-mail: liush@shutcm.edu.cn; Huiyong Shen, E-mail: shenhuiy@mail.sysu.edu.cn.

This PDF file includes:

Figures S1 to S2

Tables S1 to S3

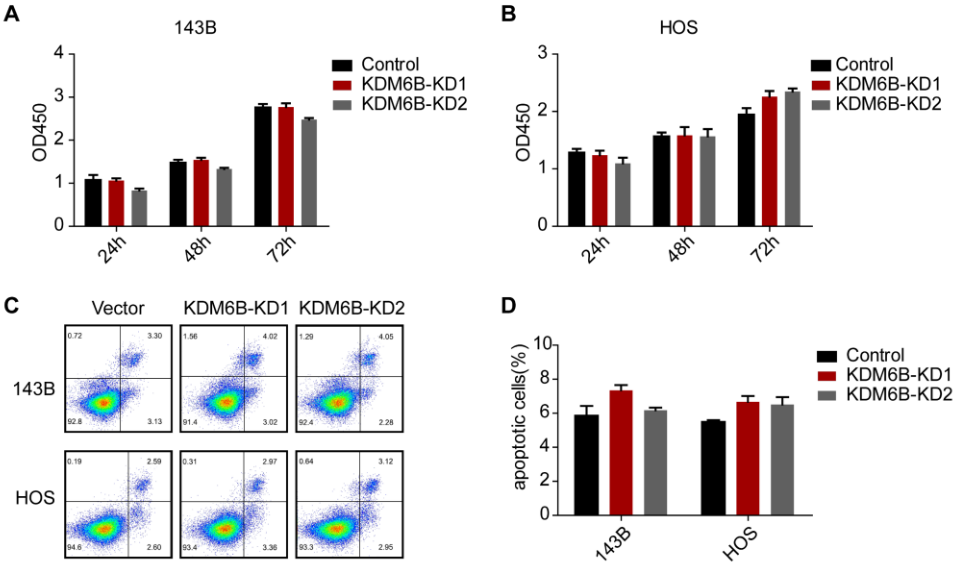

Figure S1. Intact cell growth and survival in KDM6B-KD OS cells

(A and **B)** CCK-8 assays in control and KDM6B-knockdown 143B and HOS cells.

(C) Flow cytometry profiling of Annexin-V/7-AAD-stained control and KDM6B-knockdown 143B and HOS cells.

(D) Quantitation results for (C).

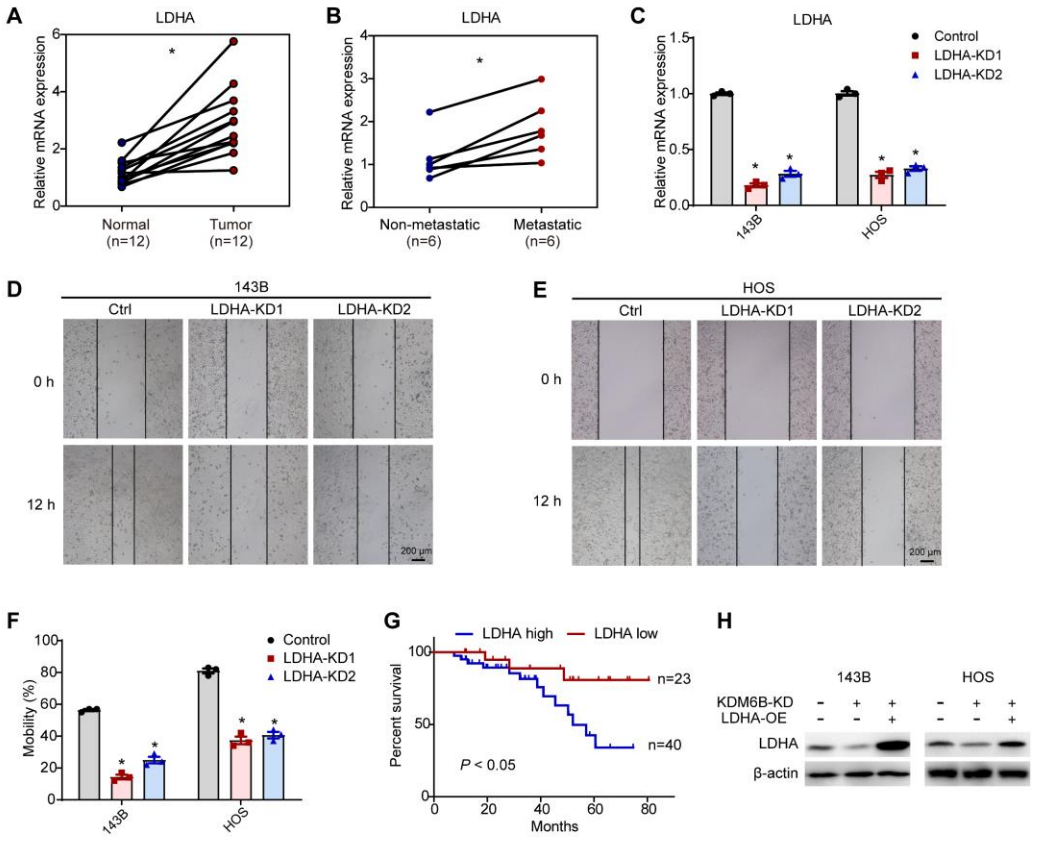

Figure S2. Elevated LDHA expression and function in OS

(A) Expression of LDHA in 12 pairs of primary OS patients determined by RT-qPCR.

(B) LDHA mRNA expression in patients with metastatic and non-metastatic osteosarcoma.

(C) LDHA mRNA expression checked by RT-qPCR in control/LDHA-KD 143B and HOS cells.

(D and E) Wound-healing assay of control and LDHA-KD 143B and HOS cells.

(F) Quantitative results for (D, E).

(G) Kaplan-Meier plot of overall survival of 63 OS patients based on LDHA expression levels. A log-rank test was used for statistical analysis.

(H) Immunoblots for LDHA expression in control/KDM6B-KD and LDHA-OE OS cells. *$P < 0.05$.

Table S1. Sequences of the RT-qPCR Primers

RT-qPCR primer	
KDM6B-F	ACCCACTGTGGTCTGTTGTAC
KDM6B-R	AATCATCACCCGTGTCTCC
LDHA-F	GGCCTGTGCCATCAGTA
LDHA-R	CAAGCCACGTAGGTCAA
VEGFA-F	GCTGCTGCAATGACGAG
VEGFA-R	GACTTATACCGGGATTTCTT
GAPDH-F	CTAAGGCCAACCGTGAAA
GAPDH-R	AGAAGGAAGGCTGGAAAA

Table S2. Sequences of ChIP-qPCR Primers

ChIP-qPCR primer	
LDHA-P1-F	TCCAGAAGCACAGCCCAGAG
LDHA-P1-R	CTTGTTCCGCATCCACCC
LDHA-P2-F	TAACAAATAGGGAGACCGAAAG
LDHA-P2-R	TGGGAGGGCATGGGATGA
VEGFA-P1-F	CGCAGCGGTTAGGTGGAC
VEGFA-P1-F	GGGGAATGGCAAGCAAAA
FGF1-P1-F	AGGCTGAAGTTTAGGATTA
FGF1-P1-R	CTGAAACGAGGATGTGAT

Table S3. Luciferase intensity of orthotopic tumor and lung metastasis

lung (cps)	Control				
Day 7	0	5	0	0	0
Day 14	15762	3382	11897	12654	3579
Day 21	102067	64056	98741	75236	113022
Day 23	660769	424373	682231	439587	502475
	shJMJD3-1 (KDM6B-KD1)				
Day 7	0	0	0	0	0
Day 14	0	0	0	0	0
Day 21	0	0	0	0	0
Day 23	0	0	722	0	0
	shJMJD3-2 (KDM6B-KD2)				
Day 7	0	0	0	0	0
Day 14	0	0	0	0	0
Day 21	0	0	0	0	0
Day 23	0	0	0	0	0
in situ (cps)	Control				
Day 7	2315	1429	123	55	87
Day 14	80330	125690	60254	72115	141451
Day 21	248488	478999	523547	178952	102479
Day 23	1502495	2813695	1724781	1925415	2712852
	shJMJD3-1 (KDM6B-KD1)				
Day 7	51	49	1239	62	118
Day 14	28590	32574	113252	64124	14126
Day 21	104212	184805	175412	95417	162415
Day 23	1851850	869488	1724784	881147	1625414
	shJMJD3-2 (KDM6B-KD2)				
Day 7	22	139	1148	24	112
Day 14	40738	74444	112365	35744	65317
Day 21	257395	148773	265412	162114	152119
Day 23	1880931	588281	1571478	1721475	624173
